# Self-compressed waveform-stable light transients enabling water-window attosecond spectroscopy

**DOI:** 10.1038/s41566-025-01802-1

**Published:** 2025-11-13

**Authors:** Valentina Utrio Lanfaloni, Federico Vismarra, Emir Ardali, Nicholas Monahan, Joss Wiese, Tristan Kopp, Fernando Ardana-Lamas, Giuseppe Fazio, Leonardo Redaelli, Yoann Pertot, Kristina Zinchenko, Tadas Balčiūnas, Hans Jakob Wörner

**Affiliations:** Laboratorium für Physikalische Chemie, ETH Zürich, Zürich, Switzerland

**Keywords:** Ultrafast photonics, Nonlinear optics, X-rays

## Abstract

The demonstration of soliton self-compression in the terawatt-level regime using large-core hollow capillary fibres and long-wavelength driving pulses has opened new possibilities for tabletop ultrafast spectroscopy experiments. Here we report the creation of phase-stable sub-cycle self-compressed light transients, as well as their field- and phase-resolved optical field sampling. We demonstrate the direct in situ measurement of self-compressed light transients, reaching durations down to 2.5 ± 0.2 fs, which is half of an optical cycle at a centroid wavelength of 1,366 nm, and determine their waveform phase offset. We apply these transients to soft X-ray high-harmonic generation and attosecond X-ray absorption spectroscopy. Attosecond transient absorption spectroscopy at 250 eV demonstrates the utility of the sub-cycle light transients for experiments with ultimate temporal resolution. The advances reported in this work merge the deep sub-cycle temporal resolution offered by self-compressed, phase-characterized light transients and showcase their application for water-window attosecond X-ray absorption spectroscopy, pushing the boundaries of achievable temporal resolution.

## Abstract (web summary)

Researchers demonstrate phase-stable sub-cycle self-compressed light transients, as well as their sampling down to half of an optical cycle, and determine their waveform phase offset. They apply the transients to soft X-ray high-harmonic generation and attosecond X-ray absorption spectroscopy.

## Main

Understanding and controlling the electron dynamics in atoms and molecules is essential to study the underlying electronic structure that defines the properties of materials and governs chemical reactions and other ultrafast processes. In the past decade, there have been significant advances in developing tabletop few-cycle sources suitable for driving strong-field processes^1^, notably creating attosecond pulses through high-harmonic generation (HHG)^2–4^ and probing electron dynamics^5–9^. However, the time resolution of most of these experiments has remained limited by the use of few-cycle infrared (IR) pulses^10,11^, acting as either the pump or the probe pulse. A promising solution to this problem has been the introduction of intense synthesized sub-cycle light transients^12^, which confine nonlinear light–matter interactions to sub-cycle durations, but their generation has so far necessitated complex experimental implementations^13–15^. The recent demonstration of soliton self-compression in large-diameter hollow-core fibres (HCFs)^16^ has created an alternative approach to obtain intense sub-cycle light transients in a robust and simple experimental set-up.

Here we report the waveform-resolved measurement of self-compressed light transients, their application to HHG and the realization of attosecond soft X-ray (SXR) spectroscopy. The combination of these breakthroughs advances SXR spectroscopy deep into sub-cycle temporal resolution and merges the latter with element specificity, site selectivity and all other advantages of X-ray spectroscopy^11,17–22^. Importantly, the field-resolved measurements are carried out in situ, that is, inside an attosecond beamline, which is crucial to establish the exact temporal profile of the light transients used in attosecond experiments. This is achieved by implementing the recently introduced ‘complete reconstruction, using ionization yield modulation, of the electric field’ (CRIME) method to retrieve their waveforms^23^, relying on the detection of time-resolved ion yields. Applying the light transients to HHG, we demonstrate how sculpting the waveform on sub-cycle timescales influences the process of HHG, showing that the minimum duration for productive SXR HHG is about one optical cycle. We then demonstrate the first realization of attosecond transient absorption spectroscopy (ATAS) at energies beyond 120 eV (refs. ^12,18^), specifically up to the water window, with light transients, establishing deep sub-cycle resolution in SXR ATAS. To achieve the highest temporal stability feasible in our experiment, which leads to deep sub-cycle resolution, we implement an in-line geometry with an interferometric split-toroidal mirror to precisely control the delay between pump and probe pulses. This set-up allows us to use the light transient as the driver for HHG to generate the SXR attosecond pulses, and simultaneously as a pump or probe pulse in ATAS measurements.

These results establish soliton self-compression as a robust and scalable method for generating intense, phase-stable, sub-cycle light transients suitable for driving strong-field processes. By demonstrating their direct application to attosecond SXR spectroscopy with in situ waveform-resolved characterization, we lay the groundwork for a new generation of attosecond time-resolved experiments. This platform opens the door to real-time tracking of electronic dynamics in complex systems with unmatched temporal resolution and electric-waveform sensitivity.

## Experimental methodology

**Fig. 1:**
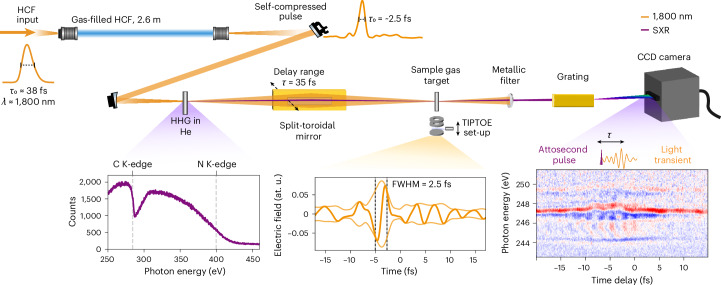
Overview of the experimental set-up. The idler beam from a white-light-seeded OPA is coupled into a 2.6-m-long HCF filled with noble gases, where spectral broadening and self-compression occur. The resulting beam is directed under vacuum into a He-filled gas cell, initiating the HHG process. The generated SXR and residual IR beams are refocused onto the sample gas target using a split-toroidal mirror. The transmitted SXR beam is dispersed by a grating and recorded on a CCD camera, with a representative spectrum shown in purple. An Al filter blocks the residual IR. The driving field for HHG is temporally characterized in situ using the TIPTOE experimental scheme in conjunction with the CRIME field retrieval algorithm. An example of a reconstructed waveform of the weak pulse is illustrated by the orange curve, demonstrating pulses broadened in 530 μm core diameter HCF filled with 2 bar of Ne. A representative transient absorption 2D map at the L_2,3_-edge of Ar reveals field-induced attosecond transient dynamics. In this case, soliton self-compression was realised in a 450 μm core diameter HCF filled with He.

**Extended Data Fig. 1:**
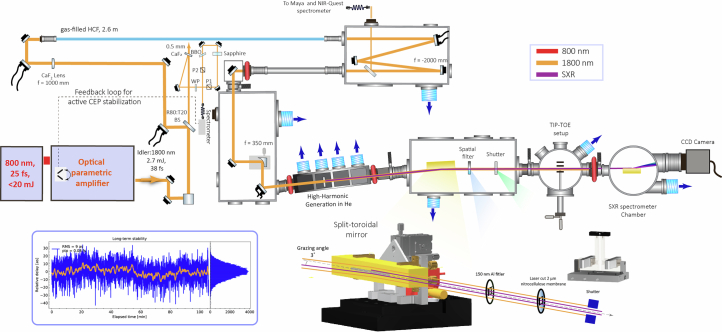
Details of the experimental setup. Schematic illustration of the experimental setup that includes the laser system (Ti:Sa laser and OPA), the 2.6 m HCF used for the generation of the light transients, the vacuum chambers housing the collimation mirror, turning mirror, and the parabolic mirror. A finite gas cell is employed for HHG in He, the split-toroidal mirror that generates and controls the pump-probe delay, and the sample interaction region where the TIPTOE setup can be moved in. The setup ends at the SXR spectrometer that includes the grating and the CCD camera. The inset shows the passive delay stability of the split-toroidal mirror. Zoom-in: split-toroidal assembly with a metallic filter to block the IR reflected from the inner mirror, a nitrocellulose membrane to suppress SXR reflections from the outer mirror, and an in-vacuum shutter that ensures the measurements without the pump. BS denotes the beam splitter that reflects 80% and transmits 20% of the idler pulses. From the transmitted portion of the BS, a Fresnel reflection of 0.5 mm CaF2 window (ca. 10%) is used for CEP characterization with an f-to-2f interferometer. A sketch of the main optical elements of the f-to-2f interferometer is illustrated.

Figure 1 shows a conceptual overview of the experimental set-ups. A more complete description is provided in Methods and Extended Data Fig. 1. We generate 38 fs pulses centred at 1,800 nm with a pulse energy of 2.7 mJ using a white-light-seeded optical parametric amplifier (OPA) pumped with 800 nm pulses. The 1,800 nm pulses are focused into an HCF filled with a rare gas. Inside the fibre, the interplay between the gas nonlinearity and the negative dispersion of the fibre leads to spectral broadening and self-compression of the pulses^16^. The resulting compressed light transients are then focused into a gas cell filled with He, where HHG occurs, reaching the water-window spectral region up to the nitrogen K-edge. After passing through the gas cell, both diverging beams, that is, the SXR and the residual light transients, are reflected off a split-toroidal mirror consisting of an inner and outer rectangular section. The centred inner section is mounted on a piezo stage that allows for nanometre-accurate positioning, while the outer section remains fixed. This toroidal mirror refocuses both beams into a second gas target, which can be used for two purposes: (i) ion-yield detection for field-resolved characterization of the HHG-driving and pump pulses using tunnelling ionization with a perturbation for the time-domain observation of an electric field (TIPTOE)^24^, and (ii) for ATAS measurements by using a downstream SXR spectrometer. In the TIPTOE measurement, the intense part of the beam, reflected from the outer mirror, drives strong-field ionization of noble gases and acts as a temporal gate. The weak part of the pulse, reflected from the central mirror, with a waveform practically identical to the intense part, modulates the ionization and thereby imprints its waveform onto the ion yield. With the CRIME algorithm, we reconstruct the electric fields of both pulses, and in the following, we report the waveform of the weak field.

## Results

**Fig. 2:**
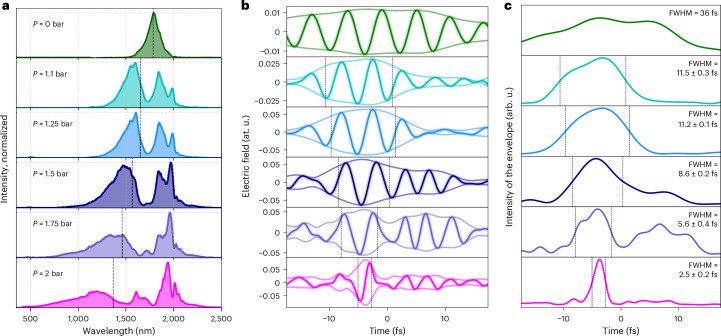
Field-resolved metrology of self-compressed light transients. **a**, Measured spectra in Ne-filled HCF of 530 μm core diameter as a function of pressure. The black dashed line shows the central wavelength of the spectrum weighted by the intensity. Going from evacuated HCF to 2 bar Ne pressure, top to bottom, they are 1,785 nm, 1,654 nm, 1,651 nm, 1,566 nm, 1,460 nm and 1,366 nm. **b**, Reconstructed electric field waveform and envelope of the weak field of pulses in vacuum and broadened in 1.1 bar, 1.25 bar, 1.5 bar, 1.75 bar and 2 bar of Ne, respectively. The temporal profiles are obtained from the TIPTOE measurements and retrieved with the CRIME algorithm. For illustration purposes, we show a representative retrieved waveform, as the CEP of the input was not actively stabilized. **c**, Intensity of the reconstructed envelope of the weak field pulses. The black dashed lines in **b** and **c** indicate the limits of the intensity envelope’s FWHM, taken as the mean value from ten independent reconstructions. The corresponding FWHM value is shown in the legend of **c**, and the error in the pulse duration is expressed as the standard deviation calculated from these ten reconstructions.

We first demonstrate the creation and field-resolved characterization of light transients, focusing on generating sub-cycle pulses. For this purpose, we chose an HCF with a 530 μm core diameter, filled with Ne gas. Figure 2a presents the measured spectra at the exit of the HCF as a function of Ne gas pressure. The spectra are corrected for the spectrometer response and normalized to the maximal intensity. As the pressure increases, self-steepening introduces spectral asymmetry, extending the broadening into the visible spectral region. This is confirmed by the central wavelength of the spectrum, which blueshifts as the pressure increases. The dashed line marks the centroid wavelength, which shifts from approximately 1,800 nm in vacuum to 1,654 nm at 1.1 bar, and to 1,366 nm at 2 bar. At a pressure of 1.5 bar, the spectrum reaches a width of about 1 octave, whereas the spectral bandwidth exceeds 2 octaves at a pressure of 2 bar.

The reconstructed electric field waveforms and the amplitude of the weak field obtained from the CRIME algorithm are shown in Fig. 2b, while the intensity of the envelope is shown in Fig. 2c. First, we note that in the case of pulses propagating through the evacuated HCF (green curves), the pulse duration cannot be reliably estimated because the measurement is limited by the scanning delay window of the split-toroidal mirror (set by design constraint at 35 fs). As a result, the reconstructed electric field does not approach zero at the boundaries of the scan range, preventing an accurate estimation of the pulse duration. Nevertheless, we can observe that the pulse length is comparable to the delay stage’s scan range. On the basis of the temporal characterization of the idler pulses before the HCF performed using frequency-resolved optical gating, and accounting for their propagation through materials before entering the HCF (5.2 mm CaF_2_, 1 mm fused silica (FS), 2.2 m of air), the duration is expected to be on the order of 36 fs.

Self-compression is achieved already at 1.1 bar, where the pulse duration decreases from approximately 36 fs to approximately 11 fs (full-width at half-maximum (FWHM)), marking a transition from a multicycle regime to 2-cycle pulses. As the pressure increases, even shorter pulses are obtained. In particular, the pressure is 2 bar, which leads to the generation of a sub-cycle light transient with an FWHM of the intensity envelope of 2.5 ± 0.2 fs. This is close to one-half of an optical cycle at the centroid wavelength of 1,366 nm (4.55 fs).

**Fig. 3:**
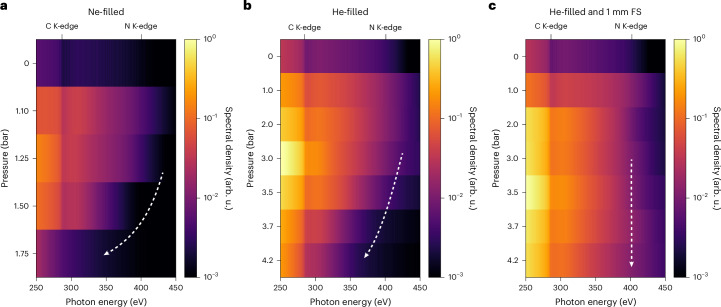
Generation of attosecond SXR pulses with self-compressed light transients. **a**, Measured HHG spectra after spectral broadening and self-compression in 530 μm core diameter HCF as a function of Ne pressure. **b**,**c**, Measured HHG spectra after broadening in 450 μm core diameter HCF as a function of He pressure without (**b**) and with (**c**) 1-mm-thick FS window inserted into the beam path before the HHG chamber. The recorded spectra were corrected for the transmission through a 150-μm-thick Al filter, the Jacobian of the wavelength-to-energy conversion, the camera’s quantum efficiency, the quantum yield and the grating efficiency. All spectra are presented on a logarithmic scale.

Having established that self-compression can create sub-cycle light transients, we now investigate their efficiency in generating SXR radiation, focusing on the water-window spectral region, specifically between 250 eV and 450 eV. Figure 3a shows HHG spectra in this spectral range generated in He using different Ne gas pressures in the HCF. The spectra have been corrected for the camera’s quantum efficiency, quantum yield and transmission through the 150 μm Al filter used to protect the detector. Given that the cut-off energy (*E*_c_) is proportional to the ponderomotive potential^25,26^, which scales with the peak intensity and the square of the central wavelength (*E*_c_ ∝ *Iλ*^2^), one might expect that shorter pulses, owing to higher peak intensity, would enhance both *E*_c_ and the photon flux. However, our findings show a more complex behaviour. The counts at the C K-edge (284.5 eV) are maximized at 1.25 bar, corresponding to 2-cycle pulses at 1,653 nm. In this case, the high-harmonic flux increases by a factor of approximately 15 compared with evacuated HCF and is estimated to be approximately 0.27 pJ per laser shot (for the energy region captured by the charge-coupled-device (CCD) camera chip). Increasing the pressure further leads to a progressive reduction of flux, and at 1.75 bar, this enhancement is reduced to approximately 1.5. Examining the cut-off behaviour, we find that the highest photon energies are achieved at 1.1 bar of Ne in the HCF. Beyond this pressure, the cut-off shifts to lower energies, and by 1.75 bar, the highest photon energies coincide with the carbon K-edge. At even higher pressures, no HHG flux is observed above the carbon K-edge, so these results are not shown here. Two fundamental contributions may be important for understanding these effects: the blueshift of the central wavelength of the driving field, which accompanies the formation of a multi-octave-spanning spectrum, and the possibility that very short transients could suppress high-energy recollision events^27^.

The influence of the central wavelength on the cut-off energy can be assessed using the intensity-weighted central wavelengths and the semi-classical model for the HHG cut-off energy^26^. Considering a peak intensity of 5.8 × 10^14^ W cm^−^^2^, as described in Methods, and given central wavelengths of 1,653 nm, 1,460 nm and 1,366 nm and durations of 11.5 ± 0.3 fs, 5.6 ± 0.4 fs and 2.5 ± 0.2 fs at Ne gas pressures of 1.25 bar, 1.75 bar and 2 bar, respectively, this results in a decrease in the cut-off energy from 495 eV to 392 eV, and further to 346 eV at 2 bar. However, this decrease in the cut-off energy is insufficient to account for the experimentally observed results.

**Extended Data Fig. 2:**
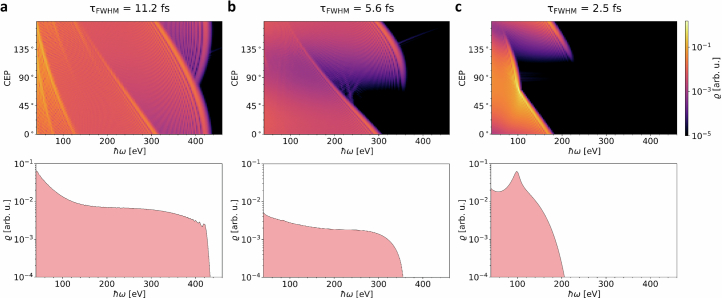
HHG spectra simulated with 1D TDSE. Top panels: simulated HHG spectra from the reconstructed electric fields illustrated in Fig. 2b as a function of phase offsets and photon energy. Bottom panels: phase-averaged HHG spectra as a function of the photon energy. Results are shown for pulses broadened in 530 *μ*m core diameter HCF filled with Ne at different pressures: 1.25 bar in **a**, 1.75 bar in **b**, and 2 bar in **c**. These pressure conditions result in driver pulses for the HHG process of durations (FWHM) of 11.2 ± 0.1 fs, 5.6 ± 0.4 fs, and 2.5 ± 0.2 fs, respectively.

To better understand the observed reduction, we thus compared the measured spectra with simulations based on the time-dependent Schrödinger equation (TDSE) shown in Extended Data Fig. 2. Detailed information on these calculations can be found in the ‘TDSE simulations’ section. Since the model does not include spatio-temporal reshaping of the driving field and phase-matching of the harmonics, it is intended only for qualitative comparison. These calculations were performed using the experimentally reconstructed field-resolved waveforms shown in Fig. 2b, maintaining a common peak intensity across all calculations. This approach aims to elucidate the effect of the pure pulse duration in the experimental observations in the HHG shown in Fig. 3a. We observe maximal cut-off energy above the nitrogen K-edge for a pressure of 1.25 bar in the HCF, which rapidly decreases with higher pressures, corresponding to shorter pulses for the HHG process. When the pulses reach the sub-cycle duration, the cut-off energy lies below the carbon K-edge, as illustrated in Extended Data Fig. 2c. Since the calculations were all performed with the same peak electric field (intensity) with averaging over all possible values of the carrier-envelope phase (CEP), the cut-off decrease observed in the calculations is clearly dominated by the reducing pulse duration, which shows that single-cycle pulses are the most efficient drivers when photon energies in the water window are desired, whereas sub-cycle pulses lead to cut-off energies that rapidly decrease with decreasing pulse durations. The main physical effect underlying these observations is the fact that deep sub-cycle transients are too short to efficiently drive both the ionization and the electron acceleration steps in the HHG process. The high nonlinearity of the ionization step requires a large electric field amplitude. For sub-cycle transients, the remaining field amplitude available to accelerate the electron is then no longer sufficient to reach the highest cut-off energies, which leads to a reduction of the cut-off.

**Extended Data Fig. 3:**
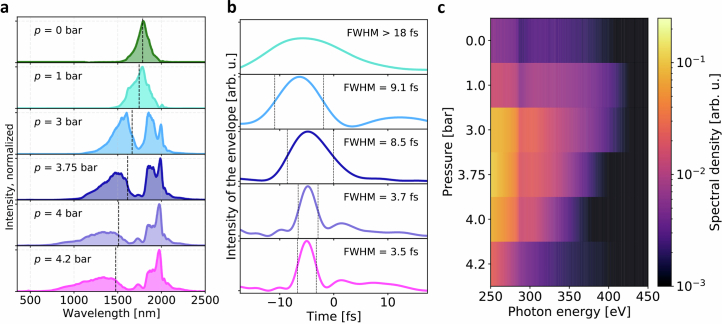
Pulse broadening and self-compression in 530 *µ*m core diameter HCF filled with He. **a** Measured static spectra at the output of the 530 *μ*m core diameter HCF as a function of He pressure. The black dashed line shows the central wavelength of the spectrum weighted by the intensity. Going from evacuated HCF to 4.2 bar He pressure, top to bottom, they are: 1,784 nm, 1,745 nm, 1,664 nm, 1,613 nm, 1,510 nm, and 1,477 nm. The spectra are corrected for the spectrometer response and normalized to the maximal intensity. **b** Intensity of the envelope of the reconstructed weak electric field waveform as a function of the He pressure, normalized to the maximum. The black dashed lines indicate the limits of the intensity envelopes’ FWHMs, obtained for a single reconstruction. **c** Measured SXR spectra generated the driving pulses for HHG characterized in panels **a** (spectrally) and **b** (temporally).

A similar trend in the high-harmonic flux and cut-off energy is observed when the pulses are propagated in He instead of Ne. Extended Data Fig. 3 illustrates, in panel a, the spectra measured after the HCF, in panel b, the intensity of the envelope from the retrieved electric field, and in panel c, the corresponding measured SXR spectra.

As the pressure in the HCF increases, progressive spectral broadening and self-compression are observed. Owing to the lower nonlinearity of He compared with Ne, higher pressures are required to achieve similarly short pulse durations. In fact, sub-4 fs pulses are obtained only at pressures exceeding 4 bar.

Focusing on the effect of these compressed pulses on the HHG, we observe a progressive blueshift of the cut-off energy, similar to the results with broadening in Ne. In addition, when the pulses become shorter than 4 fs, efficient emission of high harmonics at the nitrogen K-edge is not observed. Specifically, this is achieved at 4 bar and 4.2 bar of He, when spectral broadening and self-compression generate pulses of 3.7 fs and 3.5 fs centred at 1,510 nm and 1,477 nm, respectively. Under these conditions, an integrated energy of 0.12 pJ per laser shot and 0.04 pJ per laser shot is estimated within the spectral window of CCD detection.

The presented configurations demonstrate the power of the self-compression scheme. Starting from idler pulses of 38 fs from an OPA, we reached pulse durations of half an optical cycle and spectra spanning more than 2 octaves. However, our results also indicate that these exceptionally short light transients generate a relatively low HHG flux in the spectral region between the carbon and nitrogen K-edges.

We therefore shift our focus from reaching the shortest pulse durations to optimizing the SXR photon flux and cut-off. For this purpose, we decreased the inner diameter of the fibre to 450 μm and used He as the nonlinear propagation medium.

We now study the performance of these pulses as drivers for HHG. Figure 3b shows on a logarithmic scale the HHG spectra as a function of the He pressure in the HCF.

**Extended Data Fig. 4:**
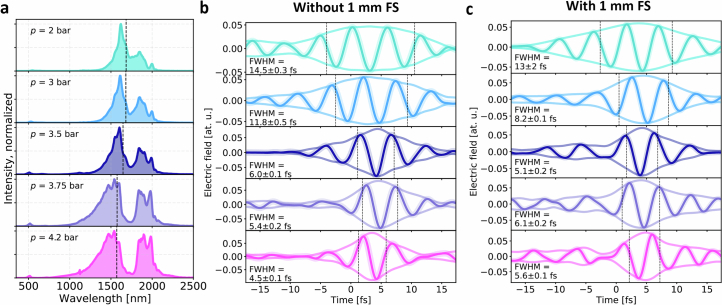
Effect of dispersive material on light transients. **a**. Measured static spectra at the output of the 450 *μ*m core diameter HCF as a function of He pressure. The black dashed line shows the central wavelength of the spectrum weighted by the intensity. Going from 2 bar to 4.2 bar He pressure, top to bottom, they are: 1,684 nm, 1,681 nm, 1,646 nm, 1,574 nm, 1,570 nm. The spectra are corrected for the spectrometer response and normalized to the maximal intensity. **b** and **c** Reconstructed electric field waveform of the weak field for the self-compressed pulses with and without 1 mm-thick window of FS inserted in the beam path before the HHG cell. The black dashed lines in **b** and **c** indicate the limits of the intensity envelope’s FWHM, taken as the mean value from ten independent CRIME reconstructions. The error in the pulse duration FWHMs is expressed as the standard deviation calculated from these ten reconstructions. In panel **c** (\SI{2}{bar}), the error was calculated from nine reconstructions, as one outlier (\SI{29}{fs}) was excluded; the others gave values between approximately 10–\SI{15}{fs}. For illustration purposes, we show a representative retrieved waveform of the electric field, as CEP was not actively stabilized.

We observe a significant overall increase in the detected photon flux compared with the 530 μm HCF filled with Ne. Specifically, the photon flux generated by pulses propagating through the 450 μm core diameter HCF filled with 3 bar of He is approximately 4 times higher than that obtained with the 530 μm HCF filled with 1.25 bar Ne at 284.5 eV. The estimated total energy of the integrated detected photon energy is approximately 1.1 pJ per laser shot at 3 bar. We attribute this enhancement to the generation of pulses at lower soliton number^28^, leading to more energy in the main envelope and less in the pedestals that intrinsically arise from the soliton self-compression, as shown in Extended Data Fig. 4. The highest photon flux is observed at 3 bar, with a driver of approximately 11 fs. With increasing pressures in the HCF, the pulses get shorter, which again leads to a decrease in the HHG flux and cut-off. Similar to the Ne-filled-HCF case (Fig. 3a), the cut-off energy is highest at 1 bar and decreases as the pressure in the HCF increases. By analogy with the Ne-filled-HCF case, this behaviour is associated with the decreasing pulse duration.

To counteract the positive chirp of a few-cycle pulse, bulk material with negative group velocity dispersion was inserted into the beam path before the HHG gas cell. Specifically, a 1-mm-thick FS window was chosen, as it exhibits negative group velocity dispersion over most of the multi-octave spectrum (with a zero-dispersion wavelength at 1.272 μm (ref. ^29^)). This addition improved the HHG flux and the cut-off energy. A comparison between Fig. 3b and Fig. 3c shows that the maximal flux is obtained at the higher pressure of 3.5 bar in the presence of FS compared with 3 bar in its absence.

We performed field-resolved measurements of the corresponding pulses, and the reconstruction, illustrated in Extended Data Fig. 4c, shows that 5.1 fs pulses are generated at a He pressure of 3.5 bar in the HCF after propagating through a 1-mm-thick FS window, corresponding to single-cycle pulses. This configuration enables high photon counts and favourable cut-off energy for the HHG spectrum while maintaining ultrashort mid-IR pulses. It also represents an optimal configuration for ATAS measurements. The ability to generate single-cycle pulses is particularly relevant for ultrafast studies involving strong-field light–matter interaction, as it confines the interaction to the single, most intense half cycle of the oscillating electric field if the waveform phase offset is approximately zero (corresponding to a cosine-type waveform).

**Extended Data Fig. 5:**
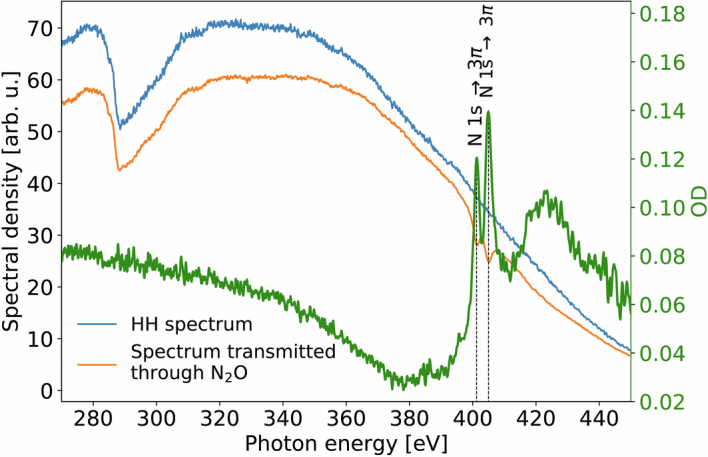
Static absorption spectrum of N_2_O at N K-edge. High-harmonic spectrum generated in He (blue curve) and spectrum transmitted through N_2_O (orange curve), both Jacobian corrected. The optical density (${\rm{OD}}=\log 10({{\rm{I}}}_{0}/{{\rm{I}}}_{\{{{\rm{N}}}_{2}{\rm{O}}\}})$) is shown in green and is calculated as the logarithm of the ratio between the reference spectral intensity (I_0_) and the intensity transmitted through ${{\rm{N}}}_{2}{\rm{O}}({{\rm{I}}}_{\{{{\rm{N}}}_{2}{\rm{O}}\}})$. The assignment of the two absorption lines is based on ref. ^42^.

To highlight the performance of our set-up in the SXR regime, we present, in Extended Data Fig. 5, a static measurement of N_2_O, demonstrating the beamline’s capability to resolve absorption spectra at the nitrogen K-edge.

**Fig. 4:**
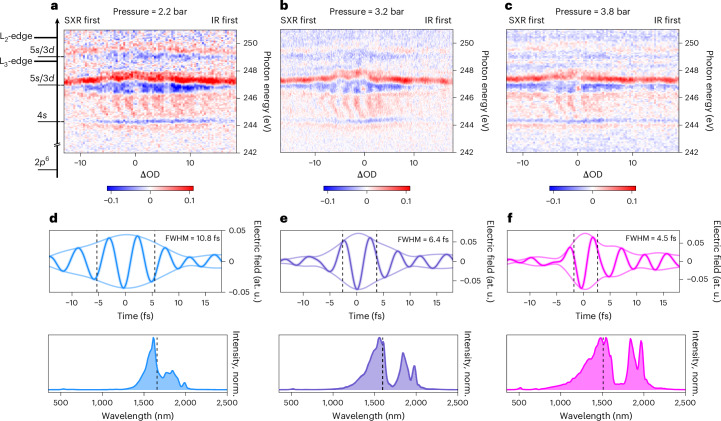
ATAS with self-compressed light transients. **a**–**c**, Measured ΔOD spectra of Ar obtained with self-compressed pulses following a 450 μm core diameter He-filled HCF at a pressure of 2.2 bar (**a**), 3.2 bar (**b**) and 3.8 bar (**c**). Reconstructed electric field waveforms of the IR pulses with CRIME algorithm (top panels) and the measured spectra (bottom panels) for the corresponding pressures, that is, 2.2 bar (**d**), 3.2 bar (**e**) and 3.8 bar (**f**), respectively. norm., normalised.

Building on our investigation of HHG with self-compressed driving pulses, we now demonstrate the application of these ultrashort light transients to ATAS experiments. Specifically, we focus on the L_2,3_-edge of Ar, whose transient absorption features provide direct access to the temporal resolution of the experiment, as previously studied in ref. ^30^. Figure 4 presents ATAS two-dimensional (2D) maps recorded for different He pressures in the HCF: (a) at 2.2 bar, (b) at 3.2 bar and (c) at 3.8 bar. These pressures were selected to investigate the effects of two-cycle (a), near-single-cycle (b) and sub-cycle (c) IR pulses on the observed dynamics.

**Extended Data Fig. 6:**
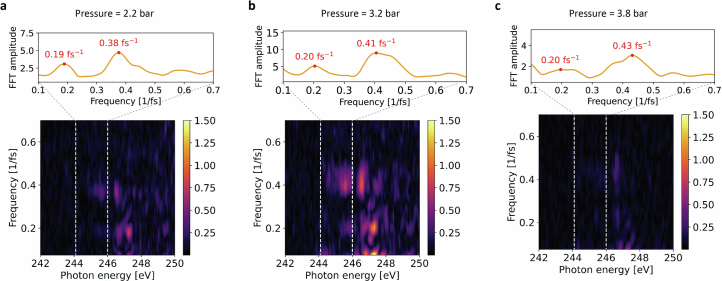
Effect of the pump self-compression on the ATAS measurements. Fourier-transforms of the ATAS measurements shown in Fig. 4, obtained with pulses that were self-compressed at three He pressures in the HCF: 2.2 bar (**a**), 3.2 bar (**b**), and 3.8 bar (**c**). The bottom row shows the Fourier maps and the top row shows their sum between the two white dashed lines.

As reported in ref. ^30^, the ATAS measurements are dominated by the AC Stark shift of two autoionizing states with the configurations $2{p}_{3/2}^{-1}4s$ and $2{p}_{3/2}^{-1}5s/3d$. The AC Stark shifts of these two levels exhibit opposite trends: the former shifts towards lower energy, while the latter shifts towards higher energy during the overlap of IR and SXR pulses. In addition, it reflects the temporal structure of the pump pulses, which is composed of a short main pulse and a longer pedestal (extending beyond the delay range accessible by the experiment). The ATAS measurements confirm the progressive temporal compression as a function of the gas pressure within the HCF of the main pulse of the pump pulses. As illustrated in Fig. 4a–c, the AC Stark shift closely follows the main envelope of the pulses. In the transient absorption maps, a broad feature appears in the spectra between 244.1 eV and 246 eV, which is attributed to the formation of light-induced states. In agreement with ref. ^30^, this signal shows 2*ω* oscillations, distinctive fringes that beat with half the period of the IR pulse. This beating is visible in all three 2D maps and reflects the blueshifting of the IR pulse’s central wavelength owing to spectral broadening of the soliton self-compression scheme. A Fourier analysis done at each photon energy and integrated over the range from 244.1 eV to 246 eV shows that the oscillation periods are 2.7 fs at 2.2 bar, 2.5 fs at 3.2 bar and 2.3 fs at 3.8 bar. Doubling these periods of oscillation corresponds to IR wavelengths of 1,618 nm, 1,498 nm and 1,391 nm, respectively. The results of the Fourier analysis are shown in Extended Data Fig. 6, where the 2D maps illustrate the fast Fourier transform at each photon energy, whereas the top panel shows the fast Fourier transforms of the signals integrated over photon energies.

The extracted IR wavelengths from the period of the 2*ω* beating exhibit a blueshift that qualitatively aligns with the intensity-weighted wavelengths determined from the measured spectra: 1,657 nm at 2.2 bar (2 cycles), 1,598 nm at 3.2 bar (1.2 cycles) and 1,510 nm at 3.8 bar (sub-cycle regime). We notice that the central wavelengths extracted from the transient signal are systematically blueshifted relative to the corresponding spectral centroids. This shift is attributed to the propagation of the IR pulses through the He gas during the HHG process. This leads to additional spectral broadening and a blueshift of the field, as previously reported in refs. ^31,32^.

**Extended Data Fig. 7:**
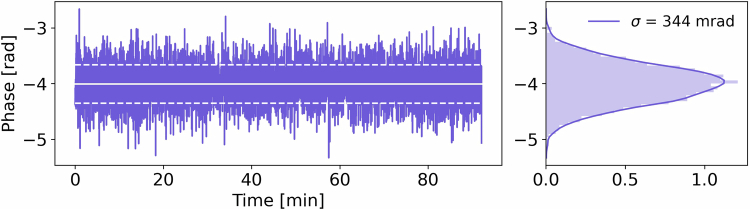
CEP stability of the input pulses during the TIPTOE measurements. CEP of the idler pulses coming out of the OPA as a function of time and its statistical analysis.

In the following, we present the reconstruction of the waveform phase offset of self-compressed light transients. Wiese et al.^23^ showed that the CRIME algorithm is sensitive to the phase of the laser-electric waveform. Building on this prediction, we show that the combination of self-compression, in situ TIPTOE technique and CRIME reconstruction algorithm can effectively retrieve the waveform phase offset of experimental sub-cycle pulses. For the realization of these measurements, we used light transients generated from a 530 μm core diameter HCF filled with 3.8 bar of He, using actively CEP-stabilized idler pulses as the input, as shown in Extended Data Fig. 7.

**Fig. 5:**
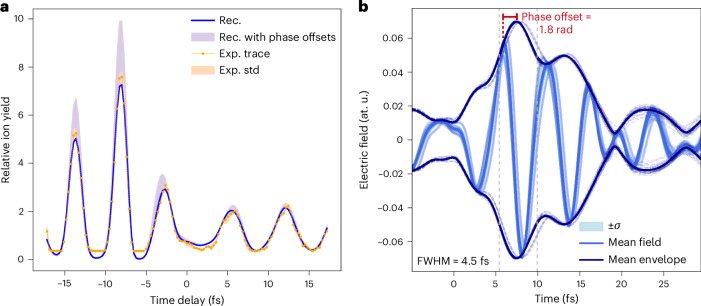
Measuring the waveform phase offset of light transients. **a**, In orange dots, the measured relative ion yield between the strong and the weak pulses; in the orange area, the standard deviation (*σ*) of the measurements. In blue, the reconstructed one. The range of variation in the relative ion yield given by the phase shifts is shown by the violet area. **b**, Retrieved waveforms from the reconstructions with *χ* value below 1.7%. In light blue, the mean reconstructed waveform is shown, while the individual reconstructions are shown in more transparent colours. In dark blue, the mean envelope of the reconstructed electric field is depicted, while results from individual reconstructions are shown with dashed lines. The vertical dashed lines show the limit of the FWHM. Rec., reconstructed, Exp., experimental, std, standard deviation.

Figure 5a shows the measured relative ion yield (orange dots), with the orange shaded area indicating the standard deviation (*σ*) of the TIPTOE experimental trace, as a function of the delay between the strong pulse and the weak pulse. The blue curve shows the reconstructed relative ion yield. To assess the degree to which the relative ion yield is sensitive to the waveform phase offset, we applied a frequency-independent phase shift to the reconstructed phase of both weak and strong pulses, ranging from 0 rad to 3.14 rad. The resulting variation range is highlighted by the violet area. The fact that this variation exceeds the experimental uncertainty confirms that the reconstruction method can determine the waveform phase offset of the measured pulses.

We performed 50 independent reconstructions of the same experimental TIPTOE trace (orange dots in Fig. 5a). From these 50 reconstructions, we selected those with a mean deviation between the experimentally recorded and the modelled relative ionization yield *χ* (ref. ^23^) below 1.7%. This criterion was set to exclude reconstructions exhibiting more than 35% variability from the optimal case, ensuring the selection of only the most consistent and reliable results. As shown in Fig. 5b, the reconstructed waveforms of the electric field are highly consistent, yielding a phase offset of 1.8 rad (defined with respect to the maximum of the envelope) with a *σ* of 0.3 rad. Since our ion-TIPTOE methodology cannot resolve the sign of the electric field, the waveform phase offset of 1.8 rad is defined modulo *π*.

Crucially, we show that these ultrashort transients can be measured in situ and in a phase-sensitive manner. This provides insight into the exact electric field driving strong-field dynamics, essential when investigating ultrafast electronic dynamics in atoms, molecules and condensed matter. The exact shape of the waveform indeed influences electronic coherences created by strong-field ionization^12^ or strong-field excitation^8^, as well as relative populations of the final states^33^. In addition, the characterization of pedestals inherent to few- and sub-cycle pulses is also a key asset for future attosecond experiments. Our approach thus combines sub-cycle temporal resolution, element specificity and field sensitivity in a flexible tabletop platform, opening opportunities for real-time studies of correlated and ultrafast electronic processes across the physical sciences.

## Conclusion

In summary, we have demonstrated the generation of light transients through soliton self-compression of 1,800 nm pulses in an HCF and their in-situ field- and phase-resolved metrology. By combining self-compression with the TIPTOE technique and the CRIME algorithm, we reported the characterization of sub-cycle self-compressed light transients, including their waveform phase offset. This capability to reconstruct the full electric field of the pulse, directly inside the attosecond beamline, represents a critical advance for waveform-controlled light–matter interaction studies, and it is essential given the high sensitivity of the self-compressed pulse to input spectral phase, energy and temporal profile.

The resulting light transients from the self-compression served as the drivers for HHG in the SXR regime (225–450 eV) and as pump pulses in an in-line ATAS scheme. This geometry minimizes temporal jitter, enabling deep sub-cycle temporal resolution in the ATAS measurements. Our approach provides the invaluable advantages of in-situ temporal characterization for measurements of ultrafast dynamics with unprecedented time resolution.

To optimize the pulse compression, we explored a range of self-compression configurations by varying the HCF core diameter and nonlinear propagation medium. We identified a configuration using Ne at 2 bar in a 530 μm core diameter fibre that produced light transients as short as 2.5 ± 0.2 fs at a central wavelength of 1,366 nm. To apply these pulses effectively in an ATAS scheme, a trade-off between the IR pulse duration and SXR flux and photon energy was identified. We optimized the configuration using a 450 μm core HCF filled with He and an additional 1 mm FS window, achieving near-single-cycle pulses while maintaining sufficient SXR photon flux.

The approach presented here bridges the simplicity and efficiency of soliton self-compression with the rigorous field-level control, required for next-generation attosecond spectroscopy. It overcomes long-standing limitations of conventional pulse compression techniques and light-wave synthesizers, providing a broadly applicable platform for tailored waveform generation. This tabletop set-up combines sub-cycle temporal resolution, element specificity through X-ray spectroscopy, and electric field and phase sensitivity. It opens the door to real-time tracking of correlated electron dynamics, light-induced states and ultrafast structural rearrangements in atoms, molecules, solids, liquids and other complex systems with attosecond precision. Looking ahead, this approach could enable a new class of experiments exploring quantum coherence, charge migration and nonadiabatic phenomena in physical, chemical and material sciences.

## Methods

### Experimental set-up

A detailed overview of the experimental set-up is shown in Extended Data Fig. 1. The core of the set-up is a cryogenically cooled 1 kHz Ti:Sa laser that pumps a white-light-seeded OPA to produce passively CEP-stable 2.7 mJ, 38 fs pulses centred at 1,800 nm. The idler beam after the OPA is split into two by an R80:T20-ratio beam splitter for s-polarized light (Altechna). The transmitted beam is directed into a second HCF, not discussed in this work. The reflected part is focused by a 1 m CaF_2_ (AR coated, EKSMA Optics) lens to a 2.6-m-long HCF, where soliton self-compression occurs. A stretched HCF (BGB Analytik AG) with a core radius of 265 μm is dynamically filled (overpressure at the entrance, vacuum at the exit) with He or Ne gas. At the entrance of the HCF, a 20 mm diameter, AR-coated, 2-mm-thick CaF_2_ window (Layertec GmbH, BBAR coated) is placed at normal incidence. The output of the HCF is directly coupled to a vacuum chamber system. The spectra at the output of the HCF are recorded using an integrating sphere (Thorlabs 2P4/M) and two ZrF_4_ optical fibres (Thorlabs MZ21L2), which separately couple into an Ocean Optics NIR-Quest grating spectrometer (for the mid-IR) and an Ocean Optics Maya 2000 grating spectrometer (for the visible spectral range). This combination facilitates the measurement of the multi-octave-spanning spectra shown in Figs. 2 and 4 and Extended Data Figs. 3 and 4. The HCF throughput depends on several factors, including the core diameter and the type of gas used for spectral broadening. For the 530 μm HCF, the throughput is about 65% under vacuum and approximately 50% with 2 bar of Ne. In He, owing to lower ionization at the input of the HCF, the transmission is approximately 64% at a pressure of 4.2 bar. By contrast, for 450 μm HCF, the transmission decreases to about 40% when filled with gas.

After the HCF, three vacuum chambers have been installed to steer the beam towards the HHG chamber. The first chamber contains an Ag mirror with a focal length of −2.0 m (EKSMA Optics) for beam collimation. The second chamber directs the beam into the focusing chamber, where a parabolic mirror with a focal length of 350 mm (LT Ultra) focuses the beam into the HHG chamber. High-order harmonics were generated in He using a cylindrical finite gas cell with a diameter of 4 mm and a length of 5 mm with a backing pressure of approximately 3 bar.

From this point, the generated SXR and the residual IR proceed collinearly until they reflect off the interferometric split-toroidal mirror. It consists of an inner rectangular mirror and an outer mirror with a central rectangular hole to accommodate the inner mirror (manufactured by Optixfab). The design exploits the different divergence properties of SXR and mid-IR radiation. The SXR beam, which has lower divergence than the mid-IR, is mostly reflected off by the inner mirror. By contrast, the outer mirror mostly reflects the more divergent mid-IR beam. The inner mirror is mounted on a piezo-electric delay stage (Physik Instrumente, PIHera P-620.1).

The entire assembly is positioned at a grazing angle of 3° to preserve the broad spectrum of the attosecond pulses and increase the reflectivity compared with normal incidence angle. A similar configuration, with a grazing angle of 15°, has been used in ref. ^34^. This set-up provides attosecond temporal resolution (referring to the inset of Extended Data Fig. 1), with a delay range of 34.9 fs.

The split-toroidal mirror also focuses both beams on the sample target with a nominal focal length of 300 mm in 2*f*–2*f* geometry.

For ATAS measurements, a 150 nm Al filter is placed directly after the toroidal mirror before the sample to suppress the residual driving field reflected off the inner mirror and avoid any IR–IR interference. An additional 150 nm Al filter is placed after the sample before the spectrometer chamber to protect the detector. The transmitted SXR beam passing through the sample is diffracted using an aberration-corrected concave grating (Hitachi 001-0659) and recorded with a CCD camera (Andor DO940P BEN). More details on the spectrometer design can be found in refs. ^35,36^.

For TIPTOE measurements, a different gas target is placed at the focus of the split-toroidal mirror. In this set-up, a time-of-flight mass spectrometer detector records the relative change in strong-field ionization yield (*Q*) as a function of the delay between an intense pulse and a much weaker perturbing pulse. The ionizing beam is reflected off the outer mirror (we refer to it as strong pulse), while the dressing beam is from the inner mirror of the split-toroidal mirror (we refer to it as weak pulse). Since the interaction of these two IR pulses is necessary for TIPTOE, the first Al filter is removed from the beam path for these measurements. The peak-fluence ratio between the ionizing and dressing beams is typically 5, for the presented measurements. The time-of-flight spectrometer follows the velocity map imaging set-up of Eppink and Parker^37^ with 10 mm spacing between the electrodes and a commercial Channeltron electron multiplier for ion detection. The signal is recorded with an oscilloscope and referenced to an electronic trigger signal from the laser. Ne is typically used for these experiments. However, He was used for the specific measurements shown in Fig. 2. The waveforms of the pulses were retrieved using the CRIME algorithm^23^.

The idler pulses used in this work were passively CEP stable^38^. The characterization of the CEP is performed with an *f*-to-2*f* interferometer built on a portion of the beam transmitted through the beam splitter. Specifically, a Fresnel reflection from a 0.5 mm CaF_2_ window (approximately 10% of the transmitted beam) is used. A half-waveplate and a polarizer are used to ensure well-defined linear polarization and intensity control of the incoming beam before white-light generation. The beam is focused into a 5-mm-thick sapphire plate to generate a white-light continuum. A 0.3-mm-thick *β*-BBO crystal (type I, 20.2°) is then used to generate the second harmonic spectral components of the broadened spectrum. The resulting interference fringes between the fundamental and the second harmonic are recorded with a Thorlabs spectrometer (CCS100). To actively stabilize the CEP, a feedback loop is implemented, linking the measured interferogram to CEP control plates located in the second stage of the OPA. The phase stability measured over more than 1 h, recorded with an integration time of 5 ms, as shown in Extended Data Fig. 7, amounts to 344 mrad (standard deviation).

### TDSE simulations

The HHG spectra presented in Extended Data Fig. 2 were simulated from the reconstructed electric fields shown in Fig. 2b, by numerically solving the one-dimensional TDSE. The model assumes a single active electron, whose Hamiltonian is described by (atomic units are used in the following if not stated otherwise)

1 $$\hat{H}(r,t)=-\frac{1}{2}\Delta +{U}_{0}(r)+r\epsilon (t).$$

Here *U*_0_(*r*) represents the field-free atomic potential and is defined as

2 $${U}_{0}(r)=-\frac{1}{2}\frac{1}{\sqrt{{r}^{2}+x}},$$

following a modified one-dimensional Coulomb potential^39^, where the parameter *x* ≈ 0.0345 was set to yield a ground state energy corresponding to the ionization energy of the helium atom (24.59 eV). The radial extent of the equidistant position grid, *r*_max_, was chosen to span twice the classical excursion length of the electron, 4*ϵ*_0_/*ω*^2^, with a position increment Δ*r* that ensured the proper description of kinetic energies up to 150% of the maximum classical recollision energy of 3.17*U*_p_:

3 $$\Delta r=\frac{\pi }{{p}_{\max }}=\frac{\pi }{\sqrt{1.5\cdot 2\cdot 3.17{U}_{{\rm{p}}}}},$$

where *U*_p_ denotes the ponderomotive energy of the electron, with typical values of *r*_max_ ≈ 510 and Δ*r* ≈ 0.45. Time propagation was performed using the Y 4-4 fourth-order $\hat{A}\hat{B}$ operator splitting scheme^40,41^, with $\hat{A}=-\frac{1}{2}\Delta$ and $\hat{B}={U}_{0}(r)+r\epsilon (t)$, using a time increment of Δ*t* = 0.1. The ground state wave function was obtained by evolving the system in imaginary time. The electric field amplitudes were scaled corresponding to a peak intensity of 5.8 × 10^14^ W cm^−^^2^ to qualitatively match the experimental cut-off energy, and—to ensure that the laser-electric field converges to zero on both sides of the time grid—a top-hat-like window function with smooth decay behaviour was applied to the reconstructed waveforms before time propagation. Finally, the time-dependent dipole acceleration was calculated using the double commutator of the position operator with the Hamiltonian,

4 $$a(t)=- < \psi (t)| \left[\hat{H},\left[\hat{H},r\right]\right]| \psi (t) > ,$$

and the HHG spectrum was obtained via the fast Fourier transform of the resulting signal. For the measurements shown in Fig. 3, the CEP was not actively stabilized. Therefore, to consider possible CEP variations in the experiment, HHG spectra were simulated considering 250 possible shifts to the CEP between 0° and 180°.

## Online content

Any methods, additional references, Nature Portfolio reporting summaries, source data, extended data, supplementary information, acknowledgements, peer review information; details of author contributions and competing interests; and statements of data and code availability are available at 10.1038/s41566-025-01802-1.

## Data Availability

Data collected and analysed during the current study are available in the ETH Data Collection, accessible via the following link: 10.3929/ethz-c-000784228.

## Code availability

The computer codes are available from the corresponding authors upon reasonable request.
